# Caring for home care workers: Health and safety hazards, harms and risk factors

**DOI:** 10.1371/journal.pone.0329959

**Published:** 2025-08-12

**Authors:** Denise M. Jepsen, Sanetta Henrietta Johanna Du Toit, Toni Robyn Barker, Saul Wodak, Erin Lawn, Tash Freeburn, Josh Rhee, Carmel Laragy, Elizabeth Convery

**Affiliations:** 1 Macquarie Business School, Macquarie University, North Ryde, New South Wales, Australia; 2 School of Medical and Health Sciences, Edith Cowan University,; 3 Centre for Disability, Research and Policy, The University of Sydney, Camperdown, New South Wales, Australia; 4 The Behavioural Insights Team, Sydney, New South Wales, Australia; 5 University of Melbourne, Victoria, Australia; UofM: The University of Memphis, UNITED STATES OF AMERICA

## Abstract

Home care workers support the growing number of older adults or people with disabilities who are living at home in their own private residences. The complex and varied work health and safety (WHS) hazards these workers face on the job include both physical and psychosocial harms. While physical WHS hazards in this sector have been articulated, there is an urgent need to update, understand and address psychosocial risks such as verbal abuse and sexually inappropriate behaviour and bullying, particularly when perpetrated by clients. This study sought to identify the range of WHS hazards and harms that home care workers face and the risk factors that mitigate or exacerbate them. Interviews with 70 stakeholders including local Australian (n = 18) and migrant (n = 17) workers and organisational providers were analyzed thematically using a hybrid of deductive and inductive coding. Common and severe hazards reported included abuse and aggression, demanding workloads, unsuitable and dynamic workspaces, manual labour, challenging client behaviours and difficulty maintaining clear professional boundaries. Few differences were identified between aged and disability sectors, or between standard and online providers. Key recommendations include improved sharing of relevant client information, addressing rather than ignoring worker isolation, promoting appropriate self-care and developing skills in dynamic risk assessment. These findings call for sector-wide reforms that integrate best practices, enhance worker support, and redefine organisational priorities to improve both worker safety and the quality of care provided.

## Introduction

Paid home care workers play a vital role in supporting older people and people with disability to live independently, yet their own work health and safety (WHS) conditions remain underexamined. Despite their essential contribution to care systems, these workers often face significant physical and psychosocial risks in the course of their daily duties. International research has drawn attention to the challenging employment and working conditions faced by home care workers globally, and recent inquiries in Australia echo many of the same concerns. The Australian Royal Commission into Aged Care Quality and Safety established in 2018 [1] and the Royal Commission into Violence, Abuse, Neglect and Exploitation of People with Disability [2] highlighted the many health and safety issues experienced by those receiving care from aged and disability services. In addition to care receivers, recent reports from the NDIS (National Disability Insurance Scheme) Quality and Safeguards Commission and the Aged Care Royal Commission highlight the varied and complex work health and safety (WHS) risks that home care workers (“workers”) face on the job. These workers are at risk of both physical and psychosocial harms.

We draw attention to the workers’ perspective and the importance of including those workers in discussions about policy development in relation to supporting their clients. We report on WHS issues faced by workers from both aged and disability sectors, based on 70 interviews with employees, employers and other stakeholders to determine their perceptions of hazards, risks and harms in this work. While important insights have been gained from research on employment conditions among fully qualified registered nurses and carers in institutional settings such as nursing homes [3], hostels [4] and assisted living [5–7], there remains comparatively less focus on the experiences of direct care workers in the home care sector. Researchers have however examined the unique and challenging work and employment conditions of home-based direct care workers in a range of countries, including Australia [8], Canada [9–14], Finland [15], Japan [16], Norway [17],Sweden [18,19], the UK [11–13], and the USA [12,20–22]. This study contributes to that literature by foregrounding the perspectives of the health and safety hazards, harms and risk factors of paid carers providing support in clients’ homes.

### Characteristics of home care workers and employment

Home care workers provide support to older adults or people with disabilities who are living at home in their own private residences, as opposed to residential care communities with dedicated staff and specialized facilities. These carers provide services including domestic duties (e.g., cooking, cleaning, gardening, maintenance), assistance with personal care (e.g., showering, toileting, dressing, giving medications), social support (e.g., accompanying clients to events, appointments or shops), assistance with mobility and transport, running errands, teaching life skills to promote independence and providing companionship [23].

The home care sector is one of the largest and fastest growing workforces in Australia [24]. As of 2021, 266,900 home aged or disability care workers were employed, projected to rise to 341,800 by 2026 [23]. Increased demands are expected in aged [25] and disability [26] services.

While there are differences in the requirements placed on the aged care and disability workforces, changes since the two royal commissions are bringing the fields closer together and there are many similarities for workers. Increasingly, workers are crossing both fields. The integration of the two fields is acknowledged by the NDIS Quality and Safeguards Commission [27] with around half the core standards in common (Table 1, quality standards in both sectors).

**Table 1:** Quality standards for Australian disability and aged care.

NDIS Practice Standards	Aged Care Quality Standards
Core standards: 1. Rights and responsibility for participants 2. Governance and operational management 3. The provision of supports 4. The support provision environment Supplementary standards: 5. High intensity daily personal activities 6. Specialist behaviour support 7. Implementing behaviour support plans 8. Early childhood supports 9. Specialised support co-ordination, and 10. Specialist disability accommodation.	1. Consumer dignity and choice 2. Ongoing assessment and planning 3. Personal care and clinical care 4. Services and supports for daily living 5. Organisation’s service environment 6. Feedback and complaints 7. Human resources 8. Organisational governance

Workers are typically older (average 47 years) and more likely to be female (80%) than the average Australian worker for all jobs (40 years, 48% female) [23]. The workforce is culturally and linguistically diverse (CALD), with around 40% born overseas [28] And mostly from Nepal, India, Philippines, England and New Zealand.

There is a range of home care worker employment models. The majority of workers are employed on a permanent, fixed term or casual basis by a traditional (or “standard”) provider of aged or disability care services who recruits and pays their own staff. Alternative employment models include: (1) being employed or contracted by a labour hire agency that is in turn engaged by an aged or disability care services provider (2) being employed or contracted by a digital platform through which clients directly request home care services from the worker (3) independent contractors who source their own clients independently of any organisation or (4) be concurrently engaged in a combination of the above types.

One third of workers are employed full-time [23], working an average of 43 hours per week, one hour less than the national average across all jobs. Although formal qualifications are not required for all home care work, 43% of workers hold the most common qualification, a Certificate III/IV in aged care, disability, mental health, community services or other related field [23].

### Worker work health and safety (WHS) experiences

Home care workers have long faced hazards on the job that can cause physical or psychological harm [29–31]. In Australia in 2020−21, ‘community and personal service workers’ had the second highest injury incidence rates nationwide, with 19.2 serious claims per thousand employees [32].

To study the behaviours and contexts contributing to WHS risks and harms in home care environments in the Australian aged care and disability services sectors, we sought to first understand and update what WHS hazards and harms these workers face and second, what characteristics compound, risk or exacerbate those risks and harms. Three WHS concepts [33] are used. A *hazard* is a situation or thing with the potential to cause harm to a person. *Risk* is a factor that increases the likelihood or severity of harm of being exposed to a hazard, while *harm* is a physical and/or psychological injury or illness which may or may not result in death. This study aimed to identify the WHS risks and harms faced by home care workers and to investigate the extent to which these risks and harms were affected by the type of service provided and the way they are engaged to perform those services. The guiding research question was ‘What is home care workers’ experience of the WHS risks and harms they face and the impact of the work context on their work duties?’

## Methods

### Participant recruitment and characteristics

Interviews were conducted with 35 organisational representatives and 35 workers. The organisational representatives were recruited through industry connections, the project’s Industry Advisory Group, plus email and LinkedIn approaches to relevant organisations. The representatives were from six key stakeholder groups (Table 2): 15 from standard providers who employ or contract home care workers to clients, four from online platform providers who employ or contract home care workers to clients, five from labour hire agencies who supply employed or contracted home care workers to providers, eight from training providers who design or deliver WHS training or tools to the sector, two from aged or disability care advocacy groups and one from a rehabilitation organisation that conducts pre-employment assessments and rehabilitation services for workers injured on the job.

**Table 2:** Number of each interviewee type, by sector (n = 35).

	Aged care only	Disability care only	Both	Total
Standard provider	6	3	6	15
Online platform provider	1	1	2	4
Labour hire agency	0	1	4	5
Training provider	4	4	0	8
Advocacy group	1	1	0	2
Rehabilitation organisation	0	0	1	1

The 35 home care workers were recruited using a targeted online Facebook advertising campaign and referrals from existing networks. Workers were incentivized with a $70 GiftPay gift card. The aged and disability care sectors were similarly represented with 12 interviewees from aged care, 17 from disability care and six who worked in both sectors. Fifteen worked for standard providers, 10 for digital providers, three who worked for both standard and digital providers, one from a labour hire agency, one who sourced their own clients, and five who did not disclose their employer-organisation type. Eighteen interviewees were born in Australia and seventeen were born overseas. Their diverse employment and demographics with respect to sex, age, CALD status are summarized in Table 3 and detailed in Table 4. Recruitment continued until data saturation was reached, with no new themes or significant insights emerging in the final interviews.

**Table 3:** Demographics of home care workers (n = 35) interviewed.

Characteristic	Detail	Interviewees
Type of care	Aged care only	12
	Disability care only	17
	Both aged and disability care	6
Gender	Female	28
	Male	7
Age	18-25	5
	26-35	12
	36-45	8
	46-55	4
	56-65	6
CALD status	Aust born, English first language	17
	Aust born, English second language	1
	Born overseas, English first language	11
	Born overseas, English second language	6
Regionality	Regional, rural, or remote	5
	Metropolitan	20
	Undisclosed	10
Organisation type	Standard provider only	15
	Platform provider only	10
	Both standard and platform provider	3
	Labour hire agency	1
	Own business	1
	Undisclosed	5

**Table 4:** Demographics of individual workers interviewed by aged and disability sector.

Care sector	Organisation type	Nature of employment	ID	Sex	Age	Aust Born	English Primary	Tenure, years
Aged	Platform	Contractor	PL_04	Male	18-25	Yes	Yes	2
	Provider	Contractor	HCW_39	Male	18-25	Yes	Yes	1
	Provider	Employee	HCW_09	Female	26-35	No	Yes	4.5
	Provider	Employee	HCW_25	Male	26-35	No	Yes	2.5
	Provider	Employee	HCW_52	Female	36-45	Yes	Yes	1.5
	Provider	Employee	HCW_53	Female	26-35	No	No	2
	Provider	Employee	HCW_77	Female	46-55	Yes	Yes	4.5
	Provider	Employee	HCW_83	Male	36-45	No	No	3
	Provider	Employee	HCW_86	Female	56-65	No	Yes	5
	Provider	Employee & Contractor	HWC_44	Female	46-55	Yes	Yes	1
	Undisclosed	Employee	HCW_73	Female	56-65	Yes	Yes	3.5
	Unknown	Employee	HCW_47	Female	46-55	Yes	Yes	5
Both – mostly aged care	Platform	Contractor	PL_02	Female	26-35	No	Yes	3
	Platform	Contractor	PL_07	Female	36-45	No	No	1.5
	Provider	Employee	HCW_87	Female	26-35	Yes	Yes	2
Both – mostly disability	Platform	Contractor	HCW_54	Female	26-35	No	No	2
	Platform	Contractor	PL_06	Female	26-35	No	Yes	2
	Provider & Platform	Employee & Contractor	HCW_28	Male	26-35	No	No	5
Disability	Independent	Contractor	HCW_61	Female	26-35	Yes	Yes	1.5
	Labour Hire	Employee	HCW_48	Female	26-35	Yes	Yes	2
	Platform	Contractor	PL_01	Female	56-65	No	Yes	10
	Platform	Contractor	PL_05	Female	56-65	No	Yes	12
	Platform	Employee	HCW_24	Female	26-35	Yes	Yes	4
	Platform	Employee	PL_03	Female	46-55	No	Yes	4.5
	Platform	Employee	PL_08	Female	36-45	No	Yes	2.5
	Platform	Employee	PL_09	Female	26-35	Yes	Yes	4.5
	Platform	Employee	PL_10	Female	26-35	No	Yes	1
	Provider	Employee	HCW_11	Female	36-45	Yes	No	2
	Provider	Employee	HCW_63	Male	18-25	Yes	Yes	1
	Provider	Employee	HCW_66	Male	46-55	Yes	Yes	20
	Provider	Employee	HCW_71	Female	56-65	No	Yes	3
	Provider	Employee	HCW_85	Female	36-45	Yes	Yes	1
	Unknown	Employee, Contractor & Provider	HCW_88	Female	36-45	Yes	Yes	4
	Unknown	Unknown	HCW_50	Female	18-25	Yes	Yes	0.5
	Unknown	Unknown	HCW_32	Female	36-45	No	No	0.5

Human research ethics committee approval (ref 2022−636) was received from Sydney University, and interviewee recruitment was conducted from 26/09/2022 to 13/12/2022. After participants emailed their written informed consent to the researchers, semi-structured virtual Zoom interviews lasting up to an hour guided by a university ethics committee-approved protocol (S1 Appendix A and B), were audio or video recorded. Questions explored experiences of WHS hazards and harms, WHS risk mitigation and exacerbation factors, and potential improvement of WHS conditions. Two experienced authors with post-graduate business and research qualifications conducted the interviews, one focusing on the questions and the other taking notes in real time. Due to commercial and privacy restrictions, the data supporting this study is not publicly available.

### Analytical approach

The study employed a qualitative descriptive approach within a constructivist research paradigm [34]. The constructivist paradigm acknowledges that participants’ experiences are shaped by their social, cultural, and organisational contexts, and that knowledge is co-constructed through the research process. Similar to other caregiver studies [14] this orientation informed both the interview design and the interpretive approach to data analysis, prioritizing the voices of workers and the meanings they assign to their experiences. The qualitative descriptive approach allowed for a rich, practice-oriented account of home care workers’ experiences with work health and safety (WHS) hazards.

Data were collected through semi-structured interviews. A hybrid approach to coding was used, combining deductive coding based on existing WHS literature with inductive coding to identify novel insights [35]. Interview transcripts, notes, and recordings were analysed thematically using a framework method, which involved coding data into a matrix in Excel, examining commonalities and differences, and generating linked themes [36]. This approach enabled both descriptive and explanatory conclusions to be drawn, capturing themes identified in the literature and those emerging directly from participant accounts.

## Results

The findings focus on themes arising from multiple interviewees, highlight notable outliers, and comparisons with the literature where appropriate. We first report the range of WHS hazards and harms, then the risk factors that exacerbate or mitigate them. The findings apply to both the aged and disability sectors unless otherwise stated.

### The range of WHS hazards and harms workers face

This section reports on the hazards and harms that workers face. The ten factors are grouped by the source of the hazard: the worker, organisation, client and environment.

### Hazard source: worker

#### 1. Physical work leading to bodily strain and injuries.

Workers are exposed to an elevated risk of physical harm due to manual handling, slips, trips and falls and excessive standing. Incidents commonly occur while cleaning, carrying groceries, moving or helping clients with bathing and dressing. Incidents often involve the performance of awkward (e.g., bending; maneuvering), high-force (e.g., lifting furniture; moving clients in and out of vehicles), tight spaces, repetitive (e.g., mopping; vacuuming), or sudden (e.g., catching clients when they fall) movements. Several providers noted that manual handling incidents were among the most common experienced by workers. Common injuries reported were musculoskeletal, including strains or sprains to the back, shoulders, knees, wrists and elbows. Several workers described ongoing soreness, with one worker noting that “aches and pains are part of the job”. This is supported by recent industry data showing that in the broader NSW healthcare and social assistance workforce, muscular stress and falls are among the top causes of workplace injuries, while soft tissue injuries and trauma to muscles, joints and ligaments are among the most common injuries [37].

Manual handling incidents sometimes occur due to improper technique [38]. Several workers described receiving no WHS training on manual handling techniques, while some providers noted that workers may often lift objects outside the scope of their work:

*“*The most common injuries we see are medium grade strains/sprains to shoulders and backs due to lifting people, instinctively catching someone instead of letting them fall, or using poor lifting techniques when picking up objects.” (*WHS manager, disability care*)

#### 2. Unclear professional boundaries leading to inappropriate workplace relationships.

Standard and platform providers and labour hire agencies all noted the nature of the job can make it hard for workers to retain a healthy social distance from their clients. Workers can form strong bonds with their clients and be motivated to provide services outside their job description or performed after hours. This can result in injury or other personal costs. For instance, one worker described buying things for clients who would promise but fail to reimburse them. Workers on digital platforms encountered a unique challenge in maintaining professional boundaries. As one female platform worker explained, new and prospective clients have direct access to a worker’s personal information such as name and photo on their platform profile. This contrasted with standard provider and labour hire contexts, where a care coordinator mediated the initial engagement between workers and clients. More than one platform provider described incidents of excessive unsolicited messages from clients to workers, and one young female platform worker’s client found her social media account. Although platforms often had inbuilt messaging features that could be monitored, clients of all provider types frequently requested the worker’s phone number or email for convenience. One worker explained that such requests were difficult to turn down:

“[Clients] can latch onto you emotionally because of the nature of the work… They’re going to form an unhealthy attachment onto the support worker, and they do have contact details.” (*Female, platform worker*)

### Hazard source: organisation

#### 3. Demanding workloads leading to physical and emotional stress.

Some workers often felt rushed with insufficient time to complete all their tasks in a shift, consistent with United Workers Union [39] findings. This occurred when the employer allocated too many duties or when clients or family members pressured them to complete extra duties. King [40] reported that organizing care work according to the principles of market logic (e.g., competitiveness, outputs), can result in a focus on care tasks being completed efficiently rather than worker safety.

One provider said staff shortages lead to fatigue because workers took on extra shifts, and demanding workloads were more common in the aged than disability sector. One aged care provider reported their workers were asked by clients’ families to perform professional cleaning services despite being outside their job scope, while another reported not having time to use the restroom or eat between client appointments. Excessive workloads were physically and emotionally draining for workers:

“[I’ve] gone grey because of work and I’m losing hair. I’ve gone to my GP and he says [it’s] stress. And it is work stress, because I’m doing the best I can but I am a one-man army” (*Female home aged care worker*)

Adamson et al. [41] proposed that although migration is a potential solution to the workforce shortfall, it is not a prominent workforce building strategy. Given that visas are primarily skills-based, migration may contribute to building workforce numbers but is unlikely to address the skills shortage [42].

#### 4. Driving-related hazards leading to potential accidents.

Driving was a regular feature of the job for many workers, who can spend substantial amounts of time on the road whether commuting to or between client homes, running errands for clients, or transporting clients to events, appointments or shops. One WHS manager described vehicles as the “workplace in between jobs”. Workers in rural or remote communities need to commute longer distances and travel on windier roads than urban workers who can experience stress and fatigue from traffic delays.

Several workers described incidents when transporting clients, such as clients attempting (sometimes successfully) to exit the car while still in motion, while another described a client attempting to grab the steering wheel during a trip. One worker described transporting clients as one of the more stressful job duties, echoing other findings (e.g., [39]).

“I had a dementia client who wanted to get out of the car while I was still driving. I now have to use child locks.” (*Female, aged care worker*)

#### 5. Isolation leading to disconnection and professional loneliness.

Consistent with prior research [43], some workers said they experienced few, if any, interactions with colleagues while at work. With limited peer interactions, experienced workers past their initial training miss out on organisational support and contact with colleagues and supervisors that occurs elsewhere. One disability worker described having no one to debrief with after shifts, and another said they would like to visit a physical office to meet other workers from time to time. The submission to the Royal Commission into Aged Care Quality and Safety by the Accord on the Remote Aged Care Workforce [44] reported that remote and regional workers experience greater separation from colleagues and employers than metropolitan workers.

Some organisations provide buddy shifts, direct support and peer networking to address worker isolation, however beyond care manager or administrative client visits, clients tended to not pay for a second worker to attend a shift. Some workers said isolation was an increasingly prominent hazard, as previously there was more access to peer support.

“Peer support is a valuable thing in this field and it is really being destroyed.” (*Male, disability care worker*)

### Hazard source: client

#### 6. Physical aggression, verbal abuse leading to physical or psychological injury.

Many workers and providers described instances of physical aggression (i.e., grabbing, hitting, biting, throwing objects) and verbal abuse (i.e., threats of violence, being sworn at). Two workers reported that triggers of verbal abuse from clients or client families were how workers were doing their job (e.g., their cooking), or workers refusing tasks outside their job scope. Consistent with Overgaard et al. [45], stakeholders reported racially charged verbal abuse of some CALD workers. One described finding it difficult to form trust and build rapport with clients after a violent outburst that caused a physical injury:

*“*The accumulation of yelling and verbal aggression over time, as opposed to acute incidents, can lead to stress and compassion fatigue.” (*WHS manager, disability care provider*)

Several workers reported clients or their family members making sexual advances towards them. Platform-based work could expose workers to a heightened risk of sexual harassment as one digital platform WHS manager described people creating fake accounts to contact workers in whom they had a sexual interest.

Abuse and aggression including distress, exhaustion and compassion fatigue have caused physical and psychological harms to workers. One provider said 60% of home aged care workers have experienced workplace abuse and aggression from clients, while United Workers Union [39] reported around 70% have felt unsafe in a client’s home. The risk of abuse or aggression is higher when caring for clients with serious mental health or behavioral issues. One labour hire agency representative said these issues are more common for disability than aged care.

*“*Physical abuse… can just come from nowhere… all of a sudden something will set them off, something in their mind.” (*Female aged c*are *worker*)

#### 7. Distressing events leading to psychological injury.

Workers reported distressing events at work that can result in psychological harm. At an extreme, the death of a client and watching families grieve can lead to disenfranchised grief [46] when others do not recognise the nature of the workers’ relationship with the client. Other distressing events include deterioration in a client’s mental or physical health, seeing clients self-harm, seeing clients living in squalor and observing client elder abuse from family members. For example, an aged care worker described finding it hard to “switch off” when their client had died, and another witnessed their client’s husband not allowing his wife to leave the house or purchase necessary items.

Workers also experience distressing events secondhand through hearing of clients’ or colleagues’ distressing experiences (e.g., when clients disclose suicidal ideation or history of abuse and neglect).

“Witnessing the mental health deterioration of clients, or clients’ suicidal ideation, can lead to psychological harm [for the worker].” (*CEO, disability labour hire company*)

One disability worker who was neglected as a child was distressed after witnessing incidents of child neglect and made a WorkCover claim to recover.

#### 8. Challenging client behaviours leading to stress or fatigue.

Clients sometimes behave in ways that are neither abusive nor aggressive but can nevertheless result in psychological harm to workers. These behaviours are typically associated with the client’s cognitive disabilities or mental health challenges and are not intentional. Situations that resulted in workers feeling stressed or fatigued included being hypervigilant for long periods to ensure clients do not wander off and put themselves in danger, having their opinions and actions repeatedly challenged by clients, being kept waiting at their client’s front door for extended periods and clients refusing to take their medication. Clients with obsessive-compulsive related disorders can assign meaningless tasks or set strict arbitrary rules for how a task should be performed (e.g., helping to hoard rubbish or being required to use a specific rag for all cleaning duties). Such tasks left workers feeling demoralized and wondering if they are reinforcing their client’s symptoms.

### Hazard source: environment

#### 9. Unsafe client homes leading to physical injury.

Lucas and Elliott [47] stress that due to its own set of safety hazards, each home is essentially a new worksite. Similarly, interviewees noted that many homes were poorly laid out and not designed to safely accommodate workers (e.g., showers unable to fit two people, or lacking fittings such as handrails). Other contributing factors included confined or cluttered spaces, rubbish obstructions in hoarders’ homes, protruding or falling objects when opening cupboard doors and glass or needles stepping hazards. One provider noted that trips and falls caused by rugs, furniture or clutter were common. Another reported a spinal injury after tripping on a garden hose left on a client’s staircase.

#### 10. Pathogens, poisons in uncontrolled workplaces leading to exposure, illness or disease.

Workers could be exposed to pathogens and poisons in an unfamiliar home. These included bodily secretions (e.g., blood, urine and faeces), drugs (e.g., cigarette smoke, marijuana and illicit injectables), diseases (e.g., COVID-19), allergens (e.g., animal fur), rubbish (e.g., dirty dishes), contaminated food offered by clients, pests (e.g., snakes and mice), mould and cleaning products (e.g., bleach), resulting in infections or asthma symptoms. Maddox [48] similarly reports on exposure to pathogens and poisons by Australian health and community workers.

Even when aware of the WHS hazards in a client’s home, workers or organisations have little power or agency to remove them. For instance, workers can find it challenging to tell a client not to smoke in their presence:

“Risks to workers include clients who smoke, which can exacerbate asthma.” (*Workers’ compensation manager, aged care*)

“In independent living, you’re always going into a work environment that’s a little bit unknown… You never know what’s happening at the house, it’s a little bit uncontrolled.” (*WHS manager, disability care*)

### Risk factors mitigating or exacerbating WHS hazards and harms

This section reports on the thirteen risk factors that mitigate or exacerbate WHS hazards. The factors are grouped by source: the worker, organisation, client and environment.

### Risk factor: worker behaviours

#### 1. Taking shortcuts.

Some workers, training providers and standard providers said workers may not always follow correct WHS techniques or protocols on the job. Taking shortcuts could exacerbate several of the WHS hazards described above. For instance, workers could neglect to change gloves between duties or clients or could physically lift a client alone when a second worker or hoist is needed. Workers take these WHS shortcuts due to pressure from clients or their family, not having enough time to perform all assigned duties safely or complacency to take shortcuts after a long time in the industry. One worker said when clients or their family saw one worker “bend the WHS rules”, they expected others to be willing to do the same.

The lack of supervisory feedback and lack of consequences for unsafe behaviour can undermine their health and safety. One worker noted “I’m sure it is not WHS approved, but no one’s seeing me, so no one’s told me off for it.”

“To do things properly sometimes would take too long.” (*Female aged care worker*)

#### 2. Prioritising client welfare over their own.

A strong desire to help their clients can put workers at risk of harm. One provider said the ‘patient always comes first’ mentality drove many risky behaviours. Workers “took shortcuts to be kind”, completing tasks they are not supposed to or deprioritizing their own WHS. Aged care interviewees spoke of prioritizing client needs more than those in the disability sector.

Several workers reported it challenging to distinguish between their own and their clients’ WHS needs. When asked about hazards or policies on worker health and safety, several described hazards or policies related to their client’s welfare, indicating they prioritized their client’s welfare potentially at the cost of their own welfare. Moreover, several providers also reported that client person-centered care was adhered to at the expense of worker safety. A managing director of an online platform provider said they are “…concerned with risks to our clients more than we are concerned to [sic] workers”, and it is the worker’s responsibility to look after WHS requirements, given they are contractors:

“The industry is very customer focused. We tend to get people putting the customer first. [Workers are] not as risk averse because… the customer needs are [seen as] greater than the risk to [themselves]… so they take on risky behaviours.” (*WHS mgr, disability care*)

“New reforms focus on client control and choice and we have lost the focus on workers.” (*Rehabilitation organisation representative*)

#### 3. Unreasonable expectations.

While many workers have difficulty refusing pressure from clients and family to perform out-of-scope requests, several report they can effectively communicate their wants and needs with clients and managers and set reasonable expectations. For example, some workers decline shifts they do not feel qualified to perform, refuse manual handling shifts if they have an injury, or choose not to work with clients known to exhibit difficult behaviours.

Some workers seek written or verbal agreement from a client regarding the specific tasks they will perform and the client’s behaviour (e.g., not smoking or drinking during the shift). Some enforce boundaries by maintaining a physical or psychological distance from their clients. One worker described using a kitchen counter as a safety barrier when a client became aggressive, while another told us they leave a client’s residence if it is not in an appropriate state or the client refuses to comply with a request.

Organisational policies could assist to enforce boundaries with clients. For example, one online platform provider said they instruct workers not to share personal phone numbers with clients, and they monitor client-worker messages for excessive numbers of messages.

“[When] clients pressure me to do many tasks in a short time… I just say no with reasons given or explain my views with asking for extra time to be paid for chores to be done.” (*Female, aged and disability care worker*)

#### 4. Distressed clients.

Some workers effectively use de-escalation techniques to mitigate the harms associated with abuse and aggression. One worker used music and talked to distressed clients about their interests. Another drew on background knowledge of a client living with dementia to calm them when distressed. At least two providers included de-escalation techniques in their training program. However, there are no de-escalation standard training techniques in the aged or disability care sectors:

“Knowing their history a bit really helps [with de-escalation]... I think that’s an important part that the industry doesn’t really recognize.” (*Female, aged care worker*)

#### 5. Cultural and language barriers.

As a substantial proportion of the home care workforce was CALD, WHS materials and processes should be readily accessible to workers from different cultural backgrounds. Bilingual workers are especially beneficial for CALD clients living with dementia who reverted to their first language. However, one provider stressed that difference in cultural customs between workers and their clients could increase the likelihood or severity of harm associated with WHS hazards. For example, many African, Filipino and Nepalese workers found it hard to say “no” when asked to complete unreasonable tasks by clients. This could lead to workers bending rules or taking shortcuts.

#### 6. Physical characteristics.

Workers’ gender, age, weight, fitness and length of service were all referred to as characteristics that could contribute to their risk of physical injuries on the job, particularly in relation to manual handling. These characteristics were linked to lower strength (e.g., being female, older, less fit, or lighter weight), lower agility, flexibility, stamina or balance (e.g., being older, less fit, or heavier weight) and more accumulated bodily wear-and-tear (e.g., being older, or longer service history). One female disability care worker (aged 56–65) described being asked to perform duties unsuitable for her back injury, despite having informed her employer she was unable to perform manual handling duties. The rehabilitation organisation interviewee added that older age, higher body mass index and lower strength were associated with poorer recovery outcomes following injury.

Even for workers with sound physical capabilities, there were risks for male and female workers when clients were bigger and aggressive:

“I’m about 45 kg myself, and having to help men shower sometimes, especially if they’re a lot bigger than you, it’s a bit weird, feels a bit awkward.” (*Female, disability care worker*)

#### 7. Quality of WHS training.

Many workers considered they did not receive adequate WHS training and that they lacked the critical skills and knowledge needed to safely navigate workplace hazards such as de-escalating abuse or aggression, handling pathogens and poisons safely and using correct manual handling techniques and equipment.

### Risk factor: organisation behaviours

#### 8. Information provided by organisations.

Workers and providers all acknowledged that workers needed to know details of the client’s background including their behavioral history, criminal record, home environment and pets, and any medical and psychological conditions. However, multiple obstacles to information gathering existed. Care plans were often not updated as circumstances changed and information was not always conveyed clearly. Advertisements posted online by clients for platform workers were often inaccurate.

One worker and one training provider emphasized the importance of involving workers in developing in-person risk assessments and care plans when clients were first engaged:

“When you’re going into someone’s place… you wouldn’t know the place at all, or if it’s a client you’ve never dealt with before… you don’t know if they’ve got any sort of other illness, or confusion, or dementia or aggression… you don’t know if there are family pets onsite.” (*Male, aged care worker*)

#### 9. Organisational support.

There were mixed reports about organisational support for workers. Some workers felt unsupported by their employer. Examples of inadequate support included a worker meeting their employer just once in two years of service, providers not being available for workers to make a report or debrief on weekends and providers not following up on reported incidents or hazards. One worker was offered shifts with a client after reporting that client made death threats towards them. Another said they no longer reported incidents because there was no or inadequate follow-up after an incident involving client violence. Further, some workers feared losing shifts if they refused work offered.

Lack of support could exacerbate many hazards including isolation, demanding workloads and psychological harms after witnessing distressing events. These findings accord with King [49], who found the primary reason for job dissatisfaction among 100 aged care workers was unsupportive management.

There were also positive reports of organisations supporting workers when they make requests or decline shifts. For example, one worker was given time off after a client passed away. Beyond the worker-organisation relationship, one labour hire interviewee said it was crucial to build strong collaborative relationships with their host employers as they had a shared duty of care and relied on information exchange to keep workers safe.

#### 10. Non-standard employment contexts.

Workers in non-standard employment (i.e., those working for labour hire agencies or online platforms or who work independently from any organisation) could be exposed to additional factors that exacerbate WHS risks and harms. One worker said that as a contractor in an independent organisation, they were less likely to receive briefing information and broader organisational support than those employed by other types of organisations. This supports research that found training opportunities are largely unavailable to self-employed platform workers more generally [50]. Contracted workers could also experience more isolation and feelings of unfamiliarity if regularly switching between clients and home environments. The level of support offered by online platform providers can also vary between providers (e.g., in level of safeguarding or vetting clients and workers). One labour hire agency said they do not hire workers out to online platform providers due to the low organisational oversight of worker welfare.

### Risk factor: client behaviours

#### 11. Clients with complex needs.

Many workers and providers reported that workers had difficulty managing the behaviours of clients with a history of violence, were heavily medicated, or had psychological conditions such as dementia, psychosis, substance use disorder, autism spectrum disorder and attention-deficit/hyperactivity disorder. Worker risks and stress increased if they were not informed about these circumstances before entering the client’s home. Physical aggression and verbal abuse were more common in clients with dementia and certain psychological conditions. These clients required sustained and hypervigilant attention, which is tiring and stressful for workers. One worker said they use alcohol to relax after shifts with complex clients. Family members also can add to worker stress. For instance, a family member refused to give a client their medication, which led the client to engage in violent outbursts and self-harming behaviours.

### Risk factor: environmental factors

#### 12. Client homes lacking safety equipment.

Beyond client homes being unpredictable and uncontrolled workplaces, those homes can lack adequate equipment for the safe performance of domestic (e.g., vacuuming) and mobility (e.g., lifting obese clients) duties. Interviewees described bathrooms without slip mats or handrails, rooms that are too small to maneuver obese or wheelchair clients without hoist access. WHS risks of home care work without adequate equipment has been previously reported [39].

Limits on a client’s funding and lengthy approval processes often resulted in inadequate safety equipment and poor maintenance. Even if extra funding could be accessed, approvals for new equipment sometimes took months. One aged and disability provider reported that some care coordinators continued to transfer clients who lack appropriate equipment to different providers until one was willing to take them on.

#### 13. Working solo.

All the risk factors described above are heightened because workers typically work alone. When an incident occurs (e.g., a physical injury or incidence of violence), working in isolation prevents or slows access to immediate support or intervention. One worker described feeling like they needed a security guard present while at work and others referred to their vulnerability of being falsely accused of abuse or aggression because they do not have any witnesses to come to their defense. Workers in rural or regional locations were further isolated when they had no mobile data or reception.

“In a sticky situation you only have your mobile phone.” (*WHS manager, disability care*)

## Discussion

These findings offer new insights into the complexity and interconnectedness of WHS risks in the home care sector. A summary of the hazards, harms and risk factors by their source is presented in Table 5. The whole of these findings adds up to more than the sum of individual risks, highlighting the systemic nature of the challenges and the interdependence of psychosocial, physical and organisational factors. While individual risks such as manual handling, client aggression and isolation have been explored and recognised [51–53], our findings present a nuanced, holistic view of how these risks are interrelated and mutually reinforcing. We show that psychosocial risks (e.g., verbal abuse, isolation, compassion fatigue) often exacerbate physical risks (e.g., improper manual handling, slips, and trips), particularly when workers are under emotional stress, fatigued or unsupported.

**Table 5:** Summary matrix of sources of hazards and risks that may lead to worker harm.

Source	Hazard: A situation or thing that has the potential to cause harm to a person	Risk: A factor that increases the likelihood or severity of harm of being exposed to a hazard	Potential harm: physical or psychological injury or illness
Worker	• Physical work • Unclear professional boundaries	• Taking shortcuts • Prioritising client welfare over their own • Unreasonable expectations • Distressed clients • Cultural and language barriers • Physical characteristics • Not properly WHS trained	• Bodily strain and injuries • Inappropriate workplace relationships
Organisation	• Demanding workloads • Driving-related hazards • Working solo	• Organisations provide inadequate information • Organisational support • Non-standard employment can result in less support and greater isolation • Working in isolation	• Physical and emotional stress • Motor vehicle accidents • Disconnection and professional loneliness
Client	• Physical aggression, verbal abuse • Distressing events • Challenging client behaviours	• Clients with complex needs	• Physical or psychological injury • Stress or fatigue
Environment	• Unsafe client homes • Pathogens and poisons in uncontrolled workplaces	• Client homes lacking safety equipment	• Physical injury • Exposure, illness or disease

This interaction between psychosocial and physical risks presents new insight into how WHS risks in the home care sector cannot be effectively addressed in isolation. For example, a worker who is mentally exhausted from dealing with aggressive clients may be more likely to make mistakes during manual handling tasks, increasing the risk of injury. This interconnectedness calls for integrated WHS solutions that simultaneously address the physical and emotional wellbeing of workers. Solutions need to be comprehensive, addressing both the immediate hazards faced by workers and underlying systemic issues that perpetuate these risks.

Perhaps surprisingly given the potential risk of direct engagement with clients without a case manager intermediary or other screening layer, the interviews did not reveal substantial differences between workers engaged by standard providers or labour hire agencies compared with online platform providers. Future research focusing on this subgroup of home carers may find that protections inherent in the platform software, personal experiences, knowledge, skills or characteristics of these workers differ in ways that enable them to assess or manage risk, or how they may approach their work differently.

Further, and despite operating under different programs and legislation, the interviews did not reveal substantial differences in the aged or disability sectors. Hazards such as physical aggression or driving tend to be less dangerous with frail aged than with disability care recipients with developmental or mental health issues, but workers remain vulnerable for the remaining risks with both aged and disabled clients and their families. Both aged and disability sector quality standards require similar organisational systems including feedback, complaints, incident management, compliance, risk, continuous improvement, governance and human resource management (NDIS Quality & Safeguards Commission, 2022). The dominant feature of both sectors is that these workers service clients in their own homes, which can and do change as their clients’ lives fluctuate. The controlled environmental stability of hospital, residential aged or disability care and the support inherent in that stability (e.g., immediate proximate supervision, supports, tools, resources) is not available in home client services in either aged or disability care. Traditional overhead supports such as supervisory, clinical and safety inspections are managed remotely in home care, generally by exception rather than as daily or otherwise routine task.

We note that responsibility for risk assessments is not a matter simply of worker vigilance but a responsibility of the provider organisation. National safety standards [33] require a person conducting a business to ensure the health and safety of workers as far as is ‘reasonably practicable’, considering the likelihood, degree of harm, what a person knows or ought reasonably to know about, and ways to mitigate those hazards, risks and harms. In terms of how workers and organisations view WHS risks and support, a key similarity is the recognition of common WHS risks such as manual handling, client aggression and psychosocial risks such as isolation.

However, there are important differences in the perceived adequacy of training and support. Organisations often believe they are providing sufficient WHS training and resources, while many workers report inadequate preparation for dealing with manual handling, de-escalation of client aggression and managing distressing situations (such as client death, violence). These divergent views suggest a need for improved communication and alignment between workers and organisations, particularly in training delivery, ongoing support and follow-up after incidents. Strengthening these areas could significantly enhance both worker safety and job satisfaction. Further research is required to determine the efficacy of different types of training, for example some suggest that language such as *concerns* or *challenges* rather than *WHS* may be more appropriate and meaningful for both clients and workers [54].

We found the risk factors interact, accumulate and compound to increase risks faced by workers. The systemic and structural factors have a downstream influence on workers, organisations, clients and environmental factors. For example, inadequate government funding and long waiting lists limit the purchase and maintenance of ergonomic equipment that can reduce lifting needs. Broad policy-level responsibilities need to be implemented at each of the sector, organisational and individual levels to ensure that the effective care given to our aged and disabled clients is provided within a safe workplace on every occasion.

We highlight four additional issues for policy and practice implementation. The first concerns sharing of client information. Workers face increased risks when they are not provided with relevant case histories due to organisational privacy concerns, for example, about the mental health diagnosis of a care recipient. An initial WHS assessment at client intake can change in the days, weeks or months after that first assessment. Care plans feature heavily in both aged and disability sectors, yet it is unclear to what extent clients, workers and organisations can agree on sharing otherwise private client information in the ever-changing workplace of a client’s home. Communication between all stakeholders that includes respect for the worker is critical [55].

Second, isolation as a major issue for home care workers, especially in remote or rural areas. Workers’ often limited interactions with peers or supervisors exacerbates psychosocial risks such as stress, anxiety, and burnout. There is particular need for more professional interaction opportunities, especially for workers dealing with traumatic or distressing events. Consistent and accessible organisational support systems and mechanisms such as regular (virtual or in-person) debriefing sessions, structured peer networking and check-ins should be prioritized to prevent psychosocial harms and promote worker wellbeing.

Third, self-care. While many workers have access to mobile devices to log their visits and concerns [56], technology is only part of the solution. At an individual level, the impact of each hazard on different workers varied greatly, as their self-care across physical, psychological, emotional, spiritual, relational and workplace domains vary [57]. For example, some workers took time off work or received counselling to recover from abuse and aggressive experiences, while others were little impacted and considered these experiences to be part of the job. Workers’ personal lives including work-family conflict will further impact their sleep and other wellbeing indicators [58] Providers of all types must recognize and prioritize the importance of self-care and promote techniques for sustainable home care careers.

Fourth, risk assessment skills training. WHS training is frequently referred to as a solution to managing or alleviating risks. While our findings support this to some degree, we suggest not enough is being done by providers to ensure workers’ safety. Our findings demonstrate that both disability and aged carers working in clients’ homes require specific risk assessment skills that includes a focus on domestic risks that may change from visit to visit. We suggest work is needed to ensure new workers entering the sector are skilled to rapidly re-assess each workplace for new potential risks before commencing work at each client home and that organisations invest, support and resource those workers so they can identify and manage those hazards and risks.

This research is necessarily limited to WHS issues and does not report the many positive stories that workers disclose when discussing their work more broadly [59]. We acknowledge the majority of home care relationships are satisfactory, positive and sustainable. We observed commendable examples of workers and organisations working together and exhibiting proper diligence. Examples of best practices include buddy systems, the use of de-escalation techniques, setting clear boundaries, peer support networks and wellbeing support services. However, these practices are not consistently applied. Encouraging wider adoption of these approaches along with more standardized and comprehensive WHS training could help mitigate the risks faced by home care workers. Expanding access to high quality tools, resources and effective processes – such as updated care plans, safe work environments, and client behavioral information – will help workers manage WHS risks more effectively across both sectors.. These examples of positive practices can form the basis for sector-wide recommendations aimed at improving worker safety and support.

This study has several limitations. While participants were drawn from a range of roles and provider types in aged and disability home care, the data reflect experiences within one national context, which may limit transferability to different regulatory or cultural settings. Although interviews were conducted until data saturation was reached, the reliance on self-reported data may introduce recall or social desirability bias. Finally, the use of qualitative methods provides depth and contextual understanding but does not allow for quantification of the prevalence of specific WHS hazards or risks.

Future research could build on these findings by exploring the prevalence and impact of psychosocial hazards in home care using quantitative or mixed-methods approaches. Longitudinal studies would be valuable to examine how workers’ exposure to WHS risks changes over time and in response to organisational or policy interventions. Comparative studies across countries or care systems could also shed light on how different regulatory, funding, and cultural contexts shape WHS outcomes. Finally, building on a review that found home care workers’ health interventions were still “in its infancy” [60], further research is needed to evaluate the effectiveness of specific strategies—such as dynamic risk assessment training or improved information-sharing protocols—in reducing harm and improving support for home care workers.

Looking to the future, the NDIS National Workforce plan [26] recognizes challenges in attracting and supporting the required 83,000 additional workers with the attitudes, behaviors, skills and knowledge to build a responsive and capable disability workforce. Their workforce capability framework recognizes the need to support the workforce with resources, research and assessment criteria to meet the sector’s requirements. Similarly, the Aged Care Taskforce [61] recognizes the need for more skilled and diverse workers to service that growing sector. It is essential that we enable both aged and disability home carers to have safe and sustainable careers.

In conclusion, around the world much vital care work is being conducted by home care workers who face multiple workplace hazards and risks that can lead to physical and psychological harms. The health of these workers is at risk of falling through policy cracks as aged and disability clients are prioritized. These findings call for sector-wide reforms that integrate best practices, enhance worker support, and redefine organisational priorities to improve both worker safety and the quality of care provided.

## Supporting information

S1 Appendix Interview guides for home care workers and organisational representatives. (DOCX)
