# Supplementary material for: Caring for home care workers: Health and safety hazards, harms and risk factors
Source: PLoS One. 2025 Aug 12;20(8):e0329959. doi: 10.1371/journal.pone.0329959 (PMC12342263; doi:10.1371/journal.pone.0329959)
Supplement: S1 Appendix — (DOCX) [file pone.0329959.s001.docx]

# **S1 Appendix A: Participant Interview Guide, Home Care Workers**

| Part 1 - General experiences (5 min) |
| --- |
| First, I’d like to ask you about the work you do as an home care worker. Can you tell me about your job? *Prompt as needed with:*   - What kinds of services do you provide? - What kinds of clients do you see? - How long does a typical shift last? - About how many hours or shifts would you work in a typical week?   *If detail is not captured by questions listed above*  I have a couple of specific questions:   - What company(ies) do you work for? What platform(s) do you use to engage clients? - Are you an employee or a contractor? - How long have you been working as an home care worker? - Which sector do you work in (aged, disability, or both)? |
| Part 2 - Perceptions and experiences of WHS risks and harms (15 min) |
| Now I’d like to ask about your thoughts about work health and safety. This means things that happen to you on the job that might cause physical injury or illness, or affect your mental health and wellbeing. Remember that you don’t need to answer anything you don’t want to. Does that sound OK?  I’d like to start by focusing on things that might cause physical injury or illness, so things like injuring your neck or back, cutting or burning yourself, or getting sick with things like the flu or COVID.  Based on your experience, what types of things might cause physical injury or illness to you on the job? Note, we’re interested in all the different things that have the potential to cause physical injury, even if you haven’t experienced it first hand.  *Prompt as needed with*: Can you describe why you think this?  Now I’d like to focus on things that might affect your mental health and wellbeing, so things like stress, feeling anxious, distressed or depressed, or experiencing bullying and harassment. These things might arise from interactions or situations at your client sites, or from things to do with your work more generally  Based on your experience, what types of things do you think might harm your mental health and wellbeing on the job? Note, we’re interested in all the different things that have the potential to cause harm to your mental health and wellbeing, even if you haven’t experienced it first hand.  *Prompt as needed with*: Can you describe to me why you think this?  Have you ever experienced physical injury/illness, or harm to your mental health and wellbeing, due to your work as an home carer?   - Can you describe what happened? - Did you continue working? - What was the response from your organisation/employer? - Do you think that the process and response you received was ‘typical’? - Were there any long-term impacts to your health and wellbeing? |
| Part 3 - Risk mitigation and exacerbation behaviours (15 min) |
| Now I’m going to ask about some of the things you and other workers do to try to protect yourselves, as well as things that workers do that might make the job more dangerous. Anything you tell me will be confidential, and we’re not judging you or any other workers. Remember that you don’t need to answer anything you don’t want to. Does that sound OK?  Is there anything you do to stop yourself getting hurt, injured, or sick while working? What sort of behaviours are these? [*Aim for an exhaustive list and prompt if participant mentions only strategies to avoid physical harms or only strategies to avoid psychological harms. Behaviours might include ‘keeping an eye out’ for certain risks or harms.*]   - Can you describe why you do these things? - What would need to change for you to do this more often?   *Prompt if the participant only discusses physical harms:*  Is there anything you do to stop yourself from being anxious, distressed or depressed around your work?   - What sorts of behaviours are these? - Can you describe why you do these things? - What would need to change for you to do this more often?   Are there things that you do or things you’ve heard other home care workers do which might be seen as risky behaviours? This might include shortcuts or things that you ‘just do’ which might mean that they’re more likely to get injured or be harmed while working?  What sort of behaviours are these? *[Aim for an exhaustive list and prompt if participant only mentions strategies to avoid physical harms or only strategies to avoid psychological harms.*]   - Why do you think people do these things? - Have you done any of these things yourself? [If yes]: - Can you describe why you’ve done this? - What would need to change for you to do things differently? |
| Demographic characteristics and wrap-up (5 min) |
| - Is there anything else you’d like to say that you haven’t had the chance to say? - Do you have any questions for me?   Now we’re close to finishing. I’m going to ask a couple of questions about you. This information will help us to understand the similarities and differences between different groups of home care workers and will not be used to identify you or discussed outside of this project.   - How old are you? What is your gender? - What country were you born in? *[If participant was born outside Australia]* How long have you been in Australia? What language(s) do you speak at home?   Thank you very much for your time today. As a thank you, we’d like to send you a $70 GiftPay electronic gift card. To do that, I just need to confirm your email address or your mobile number. What is your email address or mobile number?  Thank you. We will be sending the gift card on [DATE]. If you haven’t received your gift card by [DATE], please reach out to me and I can follow this up for you. |

**S1 Appendix B: Participant Interview Guide, Organisational Representatives**

| Part 1 - General experiences (10 min) |
| --- |
| First, I’d like to ask a little about your role:   - What is your job title? How long have you worked for this organisation? Which sector do you work in (aged care, disability, both)?   Other notes about the participant’s role or organisation:   - Can you tell me about your organisation? - What home care services do you provide? [e.g. domestic assistance, personal care, respite care, transport, etc.] - How many clients do you work with? What proportion of clients are related to home care work?  How many employees do you have? How many are home carers? - What proportion of home carers are casual/part-time/full-time? - Do you use labour hire agencies? *If yes, H*ow often? Which agencies? What if any WHS advantages or disadvantages have you experienced when using labour hire? - Do you use any workers from digital platforms? *If yes –* How often? Which services do they do? Is this client-nominated or provider-nominated? What WHS advantages or disadvantages have you experienced when engaging labour hire workers (if any)? |
| Part 2 - WHS risks and harms among home care workers (15 min) |
| I’d like to ask about specific types of services or situations that may be riskier than others   - What kinds of services do you think are riskier than others? How so? Why do you think this? *If applicable -* How does this differ between aged care and disability care? - What differences in WHS have you observed between different locations? [i.e. Differences between metro, regional, remote, very remote] - What types of clients do you think are riskier than others? Why do you think that? - Overall, what risks would you say are the most prominent among home care workers? *Prompt as needed to discuss both physical and psychological risks* - What does your organisation do to respond to these more prominent risks? |
| Part 3 - Risk prevention and response strategies (5 min) |
| Now I’d like to ask about how your organisation manages work health and safety risks among your home care workers.   - What is your understanding of the work health and safety obligations that organisations like yours have to the home care workers you employ/contract? - What kinds of activities does your organisation do to meet these obligations? *[e.g. training, PPE, WHS committee/WHS reps]* - Consider the hierarchy of risk controls (eliminate the hazard/associated risks, reduce the risk, implement administrative controls, use PPE) - Do these activities differ for different employee groups? [i.e. if the company has employees, contractors, platform workers, etc.] |
| Wrap-up (5 min) |
| - Is there anything else you’d like to say that you haven’t had the chance to say? Do you have any questions for me? Thank you very much for your time today. |
